# The Effect of a PEEK Material‐Based External Fixator in the Treatment of Distal Radius Fractures with Non‐Transarticular External Fixation

**DOI:** 10.1111/os.12837

**Published:** 2020-12-03

**Authors:** Mao Xie, Yinghao Cao, Xianyi Cai, Zengwu Shao, Ke Nie, Liming Xiong

**Affiliations:** ^1^ Department of Orthopaedic, Union Hospital, Tongji Medical College Huazhong University of Science and Technology Wuhan China; ^2^ Department of Gastrointestinal Surgery, Union Hospital, Tongji Medical College Huazhong University of Science and Technology Wuhan China; ^3^ Department of Orthopaedic Surgery Zhijiang People's Hospital Zhijiang China

**Keywords:** Biomaterials, Distal radius fractures, External fixation, PEEK

## Abstract

**Objective:**

To explore the effect of a PEEK material‐based external fixator in the treatment of distal radius fractures with non‐transarticular external fixation.

**Methods:**

There were 48 patients in this prospective comparative study. They were divided into two groups according to the materials used: the PEEK group and the titanium group. Wrist dorsiflexion, palmar flexion, pronation, supination, radial deviation, ulnar deviation, grip strength of the palm on the affected side, kneading force, Visual Analogue Scale/Score (VAS), Disabilities of the Arm, Shoulder, and Hand (DASH) score, operation time, frequency of fluoroscopy procedures, and X‐ray results were compared between the two groups. Functional recovery was evaluated at the last follow‐up according to the wrist joint evaluation criteria.

**Results:**

The baseline data were comparable between the two groups, and no significant differences were found in age, sex, fracture types (*P* > 0.05). There was no significant difference between the two groups in the results of DASH, grip strength, and recovery of pinch force and wrist function (dorsiflexion, clavicle, ulnar deviation, deviation, pronation, and supination) (*P* > 0.05). Normal limb function was achieved in the two groups of patients at an average of 6 weeks after surgery, and there was no significant difference in X‐ray examination radial height (10.60 ± 1.59 *vs* 11.00 ± 1.53, *P* = 0.687), radial inclination (1.11 ± 0.24 *vs* 1.12 ± 0.24, *P* = 0.798), volar tilt (10.33 ± 2.13 *vs* 10.00 ± 2.08, *P* = 0.660), ulnar variance (20.87 ± 3.00 *vs* 20.38 ± 3.04, *P* = 0.748), and step‐off persistence (1.73 ± 0.69 *vs* 1.68 ± 0.72, *P* = 0.425) between the two groups (*P* > 0.05). However, the operation time (54.80 ± 12.20 *vs* 85.23 ± 15.14, *P* = 0.033) and number of fluoroscopy procedures (36.93 ± 6.89 *vs* 64.77 ± 9.74, *P* = 0.000) in the PEEK group were significantly reduced compared with those in the titanium group.

**Conclusion:**

Compared with the traditional titanium external fixator, the PEEK composite external fixator has advantages, such as a shorter operation time and fewer fluoroscopy procedures when used to treat different types of distal radius fracture.

## Graphical abstract

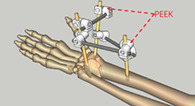

This study aimed to explore the effect of a PEEK material‐based external fixator in the treatment of distal radius fracture with non‐transarticular external fixation. We found that the PEEK composite external fixator has advantages such as a shorter operation time and fewer fluoroscopy procedures than that in the treatment of different types of DRFs compared with the traditional titanium external fixator.

## Introduction

Distal radius fractures (DRFs) occur within 3 cm of the distal part of the radius, which is the most common fracture in the upper limbs among older women and young adult males. Studies reported that DRFs accounts for 17% of all fractures and 75% of forearm fractures^1^, ^2^. Satisfactory results cannot be obtained by manipulative reduction and plaster fixation. These fractures can easily shift in position after conservative management, and complications, such as traumatic bone joint and wrist joint instability, may occur in the late stage^3^. Surgeries are performed to treat distal radius fractures so that patients can perform an adequate number of painless exercises to restore normal activity while minimizing the risk of degenerative change or disability^4^. The management of DRFs in patients aged 60 and over is performed using the following five common techniques: volar locking plate system (VLPS), non‐bridging external fixation (non‐BrEF), bridging external fixation (BrEF), percutaneous Kirschner wire fixation (PKF), and plaster fixation^5^. Patients undergoing DRF surgery with open reduction and internal fixation (ORIF) have higher risk of wound infection and tendonitis^6^. External fixators are divided into the following two types: cross‐joint and non‐bridging. A cross‐articular external fixator restricts the free movement of the wrist due to its own configuration. Nonbridging external fixators are widely used because they allow limited joint activity. Such devices can facilitate fracture reduction by fixing the fracture fragments directly; they allow easy management of soft tissue injuries and do not restrict natural wrist motion during the treatment period^7^. Therefore, nonbridging external fixators have been widely recommend for DRF treatment^8^.

In the past few decades, the use of traditional external fixators (titanium alloys) has gained popularity, because of their excellent biocompatibility, high mechanical strength and corrosion resistance^9^. However, the traditional external fixators that are made with metal or titanium may cause severe artifacts in computed tomography (CT) scans^10^, which has led to researchers looking for new materials for external fixators. Internal fixation based on polyetheretherketone (PEEK) has been studied and applied for more than 10 years. The PEEK device has the following advantages over materials used for traditional orthopaedic fixation: no metal allergies, radiopacity, low interference with magnetic resonance imaging (MRI), easier implant removal, avoiding the “cold welding” phenomenon, and better mechanical properties^11^, ^12^. For example, it has good tensile strength, bending strength, and impact strength. Some studies have shown that PEEK fixators have better strength, toughness, and stiffness than metal fixation devices, and they have better fatigue strength^13^. Although the elastic modulus of the PEEK material is 3.0–4.0 GPa, it can be strengthened by carbon fiber, and its elastic modulus can be close to that of cortical bone (18 GPa) or reach the value of titanium alloy (110 GPa) by changing the length and direction of the carbon fiber. Therefore, the mechanical properties of PEEK are close to those of bone^14^. Nowadays, the PEEK‐based external fixator has been designed and applied in clinic. However, its therapeutic effect on DRFs remain unclear.

The objective of this prospective study was: (i) to evaluate the clinical therapeutic effect of PEEK‐based external fixators on DRFs; (ii) to compare the clinical and radiological results of PEEK‐based and titanium‐based external fixators in the treatment of closed DRFs; (iii) to compare the long‐term follow‐up outcome of PEEK‐based and titanium‐based external fixators in the treatment of closed DRFs.

## Materials and Methods

### *Baseline Data*

In this retrospective study, 48 patients who underwent surgical treatment for DRFs at the Department of Orthopedics and Traumatology, Union Hospital of Wuhan, from July 2016 to September 2019, were included. The fracture types were classified according to the AO/ASIF classification system^15^. Twenty‐eight cases of different types of fractures were divided into the following two groups according to the materials used: (i) the PEEK group (PEEK material) comprising of 25 patients (12 males, 13 females, aged 31–79 years, average age 58.27 ± 11.95 years); and (ii) the titanium group (traditional titanium) comprising of 23 patients (12 males, 11 females, aged 42–71 years, average age 59.96 ± 8.29 years). Two different material‐based external fixators were selected in the two groups of patients, and they were followed up for 6 months to 1 year after the operation. All of the patients met the inclusion and exclusion criteria.

### *Inclusion and Exclusion Criteria*

Inclusion criteria: (i) DRF patients hospitalized in the orthopaedics department of our institution from July 2016 to September 2019; (ii) patients who underwent external fixators (PEEK material or traditional titanium) treatment; (iii) Visual Analog Scale (VAS), Disabilities of the Arm, Shoulder, and Hand (DASH) score, and clinical results were compared; (iv) the related outcomes of patients were completely recorded; and (v) a retrospective cohort study.

Exclusion criteria: (i) patients whose imaging findings were not consistent with the symptoms and signs of DRFs; (ii) patients who have a congenital anomaly and a pathological fracture other than an osteoporotic fracture; (iii) patients with a Gustilo–Anderson type‐3 fracture; (iv) patients with severe injury or dysfunction of major organs, coagulation dysfunction, or immune dysfunction associated with major organs; and (v) patients with upper limb disease, cancer, or other serious systemic diseases, or who were lost to follow‐up.

All of the participating subjects were fully informed about the advantages and disadvantages of both treatments, and they signed an informed consent form before the study. Approval for the study was obtained from the local ethical committee. Functional assessment was carried out by the professional staff.

### *Grouping and Definition*

**Fig 1:**
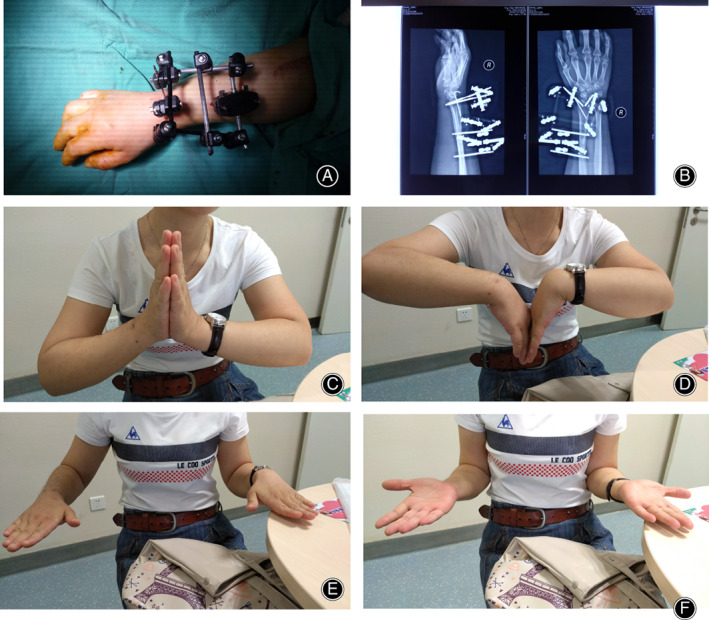
Radiographic evaluation post‐operation and recovery of wrist function at final follow‐up (PEEK group). (A) Schematic diagram of the PEEK material fixed on the distal radius fracture; (B) Radiograph of the PEEK material postoperative; (C) Wrist bending; (D) Wrist stretching; (E) Forward revolving; (F) Backward revolving.

**Fig 2:**
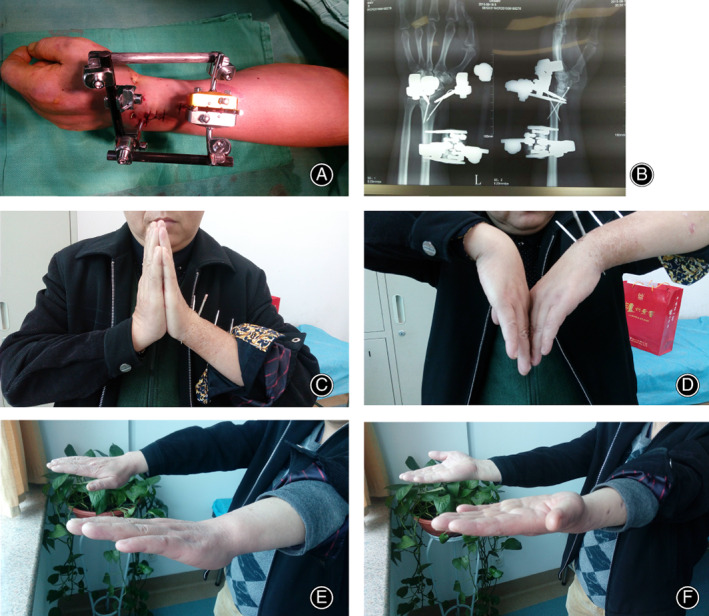
Radiographic evaluation post‐operation and recovery of wrist function at final follow‐up (Titanium group). (A) Schematic diagram of the traditional titanium fixed on the distal radius fracture; (B) Radiograph of the traditional titanium postoperative; (C) Wrist bending; (D) Wrist stretching; (E) Forward revolving; (F) Backward revolving.

We matched the patients for sex, age, and other factors, and the patients were divided into the PEEK group (PEEK material) (Fig. 1) and titanium group (traditional titanium) (Fig. 2). The patients underwent percutaneous minimally invasive osteosynthesis with one of the two materials. The patients in the PEEK group received PEEK external fixation, and the patients in the titanium group received titanium external fixation. The operation time was defined as from the beginning of anesthesia to the end of the operation. The radiographic time was defined as the total time of the C arm X‐ray machine works during the operation.

### *Surgical Procedure*

#### *Anesthesia and Position*

The patient was under brachial plexus block or general anesthesia in a supine position, and an upper limb sterilization tourniquet was applied after they were sterilized.

#### *Approach*

Closed reduction was performed under a C arm X‐ray machine, and to insert the Kirschner needle, we selected a 0.5 mm percutaneous approach at the distal ends of the radial styloid process and the radial inner column.

#### *Exposure*

Two 3‐mm pins were placed: one through the radial styloid process and the other through the distal end of the internal column. And the two proximal 4.0‐mm pins were placed through open approach between the brachioradialis. The rods and blocks were mounted, traction was applied, and the procedure was performed under radiographic control with closed reduction after the implant was attached to main bone fragments. If necessary, one or two 1.6‐mm Kirschner wires could be added to augment the stability which allowed mobility of the wrist and enabled secure stabilization of the fracture.

#### *Inspection and Dressing*

Intraoperative fluoroscopy and postoperative radiography were performed on the wrist to examine the operation. After the skin was stitched, the auxiliary dressing was bandaged.

#### *Postoperative Rehabilitation*

Antibiotics were administered prophylactically 30 min before surgery^16^. Regular dressing of the wound and sterilization of the needle were performed every day. From the end of the second day of the operation, patients were instructed to perform flexion and extension exercises of the digital joints, flexion and extension exercises of the wrist joint, and flexion and extension exercises of the ulnar muscles, elbow, and shoulder joints. The patients were instructed to visit the hospital once every 2 weeks, and the external fixator was removed during the sixth week. The patients' wrist function and DASH scores were evaluated at 6 months after surgery.

### *Observation Index*

#### *Wrist Movements and Grip Strength*

Flexion describes the movement of bending the palm down, towards the wrist; extension describes the movement of raising the back of the hand; supination describes the movement of rotating the forearm into a palm up position; pronation describes the movement of rotating the forearm into a palm down position; ulnar deviation, otherwise known as ulnar flexion, is the movement of bending the wrist to the little finger, or ulnar bone, side, which, with the right hand, is the movement you use when hitting the Enter key; radial deviation, otherwise known as radial flexion, is the movement of bending the wrist to the thumb, or radial bone, side; The measurement of wrist movements and grip strength was performed using a goniometer and hydraulic grip dynamometer. Distal radius fractures are likely to impair wrist function, and it is necessary to measure wrist movement and grip strength.

#### *Wrist Pain Score*

The VAS method is to use a sliding scale 10 cm long, with 0 indicating no pain and 10 representing the most intense pain. This method is relatively objective and sensitive to the assessment of patients' pain, and is rarely affected by other factors. It is widely used in clinical practice.

#### *Wrist Function Score*

The wrist function Krimmer scale mainly reflects the objective indexes of the doctor's examination and reflects the actual wrist function status of patients. The total score of 80–100 is excellent, 65–80 is good, 50–65 is medium, 0–50 is bad. The DASH is a standardized outcome measure that capture the patient's perspective of their status. It can reflect the impact of a disorder in terms of physical function and symptoms, which are the two main reasons patients seek care for a disorder of the musculoskeletal system.

#### *X‐Ray Measurement*

X‐rays were performed at 1 month after the operation, and the observation indexes, including radial inclination, radial height, volar tilt, ulnar variance, and articular and step‐off persistence, were compared between the two groups.

#### *Radial Inclination*

Radial inclination refers to the included angle between the vertical line of the longitudinal axis of the radius and the line connecting the furthest point of the ulnar and radial side of the distal end of the radius on the anterior and posterior X‐ray film of the wrist joint. It is meaningful to determine the reduction of the distal radius. Radial height (also known as radial length) was also measured as the distance between two lines drawn perpendicular to the radial diaphysis. Measurement of radial height can be used to assess radial shortening due to impaction or displacement, and it is an important aid in quantifying the magnitude of distal radial post‐fracture deformity.

#### *Ulnar Variance*

Ulnar variance was measured as the distance between a line drawn perpendicular to the articular surface of the ulna and the articular edge of the radius at the volar ulnar border. Volar tilt was determined on the lateral view by measuring the angle formed by a line tangential to the most volar and dorsal edge of the radial articular surface and one drawn perpendicular to the longitudinal axis of the radial diaphysis^17^.

#### *Step‐Off*

Step‐off refers to the disappearance of the normal structure of the wrist joint surface due to fracture, which limits the flexion and extension of the wrist joint, and the standard posteroanterior and lateral radiographs were used to measure articular step‐off^18^.

### *Statistical Analysis*

Statistical analysis was performed using SPSS version 19 (SPSS Inc., Chicago, IL, United States). The mean and standard deviation (x ± S) of the continuous variables (age, operation time, etc.) were assessed using the *t* test, and the categorical variables (gender, types, etc.) were assessed as a percentage (%) by using the *χ^2^* test. After performing the Kolmogorov–Smirnov test to verify that the variables were normally distributed, we applied parametric tests. A *P* value of less than 0.05 (5%) was considered to indicate a significant difference between the variables.

## Results

### *Follow‐Up*

Surgeries were successfully completed in all patients. Patients in the both group were evaluated at a mean follow‐up of 14.7 months (range, 11–18 months), and none of the patients were lost to follow‐up.

### *General Results*

We retrospectively analyzed the information of 48 patients hospitalized for the treatment of distal radius fractures from July 2015 to September 2018. Table 1 summarizes the demographic characteristics of the patients. No significant differences were found in age or sex between the two groups (*P* = 0.921, *P* = 0.773). Fractures were classified by a single experienced orthopaedic surgeon according to the AO/ASIF classification system, with the PEEK group (PEEK material) comprising of 25 patients (B1, 2 cases; B2, 2 cases; B3, 4 cases; C1, 8 cases; C2, 5 cases; and C3, 4 cases) and the titanium group (traditional titanium) comprising of 23 patients (A2, 5 cases; and A3, 4 cases; B1, 2 cases; B2, 2 cases; B3, 4 cases; C1, 8 cases), and the two groups of patients comparable in fracture classification (*P* = 0.965). The median operative time was shortened by 55.5% in the PEEK group (54.80 ± 12.20 *vs* 85.23 ± 15.14, *P* = 0.033) and the radiographic time was significantly shorter in the PEEK group (36.93 ± 6.89 *vs* 64.77 ± 9.74, *P* < 0.01). There was no significant difference between the two groups in terms of recovery (6.71 ± 0.69 *vs* 6.85 ± 0.59, *P* = 0.176) (Table 2).

**TABLE 1:** Baseline clinical data and hospitalization index

	PEEK group (n = 25)	Titanium group (n = 23)	Statistic	*P* value
Age	58.48 ± 9.62	59.96 ± 8.29	−0.567	0.921
Gender			0.083	0.773
Male	12 (48.0%)	12 (52.2%)		
Female	13 (52.0%)	11 (41.8%)		
Types			0.971	0.965
A2	5 (20.0%)	6 (26.1%)		
A3	4 (16.0%)	3 (13.0%)		
B1	2 (8.0%)	1 (4.3%)		
B2	2 (8.0%)	3 (13.0%)		
B3	4 (16.0%)	4 (17.5%)		
C1	8 (32.0%)	6 (26.1%)		

**TABLE 2:** Therapeutic outcomes of the two kinds of external fixators in the treatment of distal radius fractures (*x* ± *s*, *n* = 48)

	PEEK group (*n* = 25)	Titanium group (*n* = 23)	Statistic	*P* value
Operation time (min)	54.80 ± 12.20	85.23 ± 15.14	−8.816	0.033
Number of fluoroscopy procedures	36.93 ± 6.89	64.77 ± 9.74	−4.601	0.000
Recovery time (*w*)	6.71 ± 0.69	6.85 ± 0.59	0.896	0.176

### *Wrist Movements and Grip Strength*

There were no statistically significant differences between the two groups in terms of extension (53.40 ± 5.35 *vs* 53.09 ± 5.26, *P* = 0.757), flexion (51.36 ± 8.79 *vs* 50.48 ± 8.08, *P* = 0.699), pronation (71.56 ± 6.67 *vs* 70.00 ± 6.15, *P* = 0.331), supination (71.16 ± 5.65 *vs* 71.16 ± 4.37, *P* = 0.235), radial deviation (18.55 ± 1.37 *vs* 18.55 ± 1.31, *P* = 0.957), ulnar deviation (30.47 ± 2.63 *vs* 29.75 ± 2.82, *P* = 0.682), grip strength (41.2 ± 3.78 *vs* 41.31 ± 3.40, *P* = 0.937), key pinch (KP) (9.35 ± 3.19 *vs* 9.15 ± 2.99, *P* = 0.866)(Table 3).

**TABLE 3:** Comparison of extension, flexion, radial deviation, ulnar deviation, pronation, supination, grip strength, key pinch (KP), VAS, and DASH in the two groups (*x* ± *s*)

	PEEK group (n = 25)	Titanium group (n = 23)	Statistic	*P* value
Extension (°)	53.40 ± 5.35	53.09 ± 5.26	0.919	0.757
Flexion (°)	51.36 ± 8.79	50.48 ± 8.08	0.702	0.699
Pronation (°)	71.56 ± 6.67	70.00 ± 6.15	0.709	0.331
Supination (°)	71.16 ± 5.65	71.16 ± 4.37	0.217	0.235
Radial Deviation (°)	18.55 ± 1.37	18.55 ± 1.31	0.485	0.957
Ulnar deviation (°)	30.47 ± 2.63	29.75 ± 2.82	0.996	0.682
Grip strength kg	41.2 ± 3.78	41.31 ± 3.40	0.742	0.937
Key pinch kg	9.35 ± 3.19	9.15 ± 2.99	0.806	0.866
VAS	5.88 ± 1.07	6.13 ± 0.55	0.564	0.433
DASH	27.06 ± 10.75	26.69 ± 9.31	0.550	0.924

DASH, Disabilities of the Arm, Shoulder, and Hand; VAS, Visual Analogue Scale/Score.

### *Wrist Pain and Function Score*

There were no statistically significant differences between the two groups in terms of the VAS scores (5.88 ± 1.07 *vs* 6.13 ± 0.55, *P* = 0.433) and DASH scores (27.06 ± 10.75 *vs* 26.69 ± 9.31, *P* = 0.924)(Table 3). The consistently excellent Krimmer scores of the wrist joints in the two groups after the operation were not statistically significant (60.0% *vs* 56.5%, *P* = 0.875, Table 4).

**TABLE 4:** The excellent rate of Krimmer score of the wrist joint in the two groups after the operation

Group (*n*)	Excellent (*n*)	Good (*n*)	Commonly (*n*)	Bad (*n*)	Excellent rate (%)
PEEK group (*n* = 25)	12 (48.0)	3 (12.0)	6 (24.0)	4 (16.0)	15 (60.0)
Titanium group (*n* = 23)	9 (39.1)	4 (17.4)	5 (21.7)	5 (21.7)	13 (56.5)
Statistic	‐	‐	‐	‐	0.691
*P*	‐	‐	‐	‐	0.875

### *Radiographic Outcomes*

None of the patients were lost to follow‐up. All of the fractures were united within 2 months after surgery. Radial height (10.60 ± 1.59 *vs* 11.00 ± 1.53, *P* = 0.687), radial inclination (1.11 ± 0.24 *vs* 1.12 ± 0.24, *P* = 0.798), volar tilt (10.33 ± 2.13 *vs* 10.00 ± 2.08, *P* = 0.660), ulnar variance (20.87 ± 3.00 *vs* 20.38 ± 3.04, *P* = 0.748), and step‐off persistence (1.73 ± 0.69 *vs* 1.68 ± 0.72, *P* = 0.425) were not significantly different between the two groups (Table 5).

**TABLE 5:** Radiographic outcome

	PEEK group (*n* = 25)	Titanium group (*n* = 23)	Statistic	*P* value
Radial height	10.60 ± 1.59	11.00 ± 1.53	−0.560	0.687
Radial inclination	1.11 ± 0.24	1.12 ± 0.24	−0.138	0.798
Volar tilt	10.33 ± 2.13	10.00 ± 2.08	0.315	0.66
Ulnar variance	20.87 ± 3.00	20.38 ± 3.04	0.336	0.748
Step‐off	1.73 ± 0.69	1.68 ± 0.72	−0.111	0.425

## Discussion

DRFs are the most common type of fractures in the upper extremities, and they can lead to loss of radial inclination, radial height, palmar tilt, and a change in ulnar variance^19^, ^20^, ^21^, ^22^. Blood supply and fixation are the basic requirements for fracture healing, and the pressure stress at the end of the fracture is the key to shorten the healing time required. There is still a dispute regarding the choice of method for clinical treatment of DRFs^23^, ^24^. External fixators have many advantages in the treatment of DRFs. First, they hardly affect blood supply around the fracture ends, which is conducive to recanalization of bone vessels and creates a good fracture healing environment. Second, non‐cross‐joint fixation allows normal movement of the wrist, diminishes stiffness, stimulates cartilage repair, decreases osteopenia of the distal fragment, and reduces fear among patients^15^, ^25^, ^26^, ^27^. External fixation for the treatment of closed DRFs is widely accepted, and it is simple and effective. The materials used in external fixators include traditional titanium and new PEEK materials. PEEK resin is a specially engineered plastic with excellent performance. It has obvious advantages compared with other specially engineered plastics, including resistance to temperatures as high as 260°, excellent mechanical properties, good self‐lubrication, chemical corrosion resistance, flame retardancy, peel resistance, abrasion resistance to nitric acid and concentrated sulfuric acid, and superb mechanical properties. Therefore, this material can be used in high‐end machinery and aviation technology. A carbon‐PEEK plate can help the surgeon to obtain a good reduction because of its transparency in an X‐ray. This characteristic allows monitoring of fracture healing through visualization of callous bone. It also allows early identification of suboptimal reduction as performed by the surgeon^28^, ^29^. It is more effective for reducing the frequency of fluoroscopy procedures and the operation time, which are beneficial to the recovery of patients.

In our study, the basic indicators of the two groups and extension, flexion, radial deviation, ulnar deviation, pronation, supination, grip strength, and KP parameters were compared. There was no significant difference in the results between the two groups (*P* > 0.05). The average DASH score was 27.1 in the PEEK group and 26.7 in the titanium group (*P* > 0.05). Patients in the two groups were able to resume normal daily life activities after the operation. The average time in the PEEK group was 5.7 weeks, and the average time in the titanium group was 6 weeks (range, 2–8 weeks). Although the average time in the PEEK group was less than that in the titanium group, there was no statistically significant difference. The average VAS score was 5.9 in the PEEK group and 6.1 in the titanium group (*P* > 0.05). There was no significant difference between the two groups. The average operative time was 54.8 min in the PEEK group and 85.2 min in the titanium group, and the difference between the two groups was statistically significant (*P* < 0.05). The average number of fluoroscopy procedures was 18.7 in the PEEK group and 39.9 in the titanium group, and the difference between the two groups was statistically significant (*P* < 0.05). The results indicate that PEEK material can significantly shorten the operative time, reduce intraoperative fluoroscopy times, reduce the radiation dose, and accelerate postoperative recovery in patients. The excellent wrist Krimmer score rates after the operation were not significantly different between the two groups. These rates were 60.0% in the PEEK group and 56.5% in the titanium group. There was no significant difference between the two groups, which indicates that the two kinds of materials have no obvious effect on wrist joint function recovery. Upon X‐ray examination, there was no significant difference in radial inclination, radial height, volar tilt, ulnar variance, or articular and step‐off persistence (*P* > 0.05).

PEEK is stable, non‐toxic, and harmless. It has excellent mechanical strength and its elastic modulus is close to that of cortical bone. It can also allow X‐rays to penetrate, which is beneficial for post‐operative review^30^. This is one reason why we chose the PEEK material for this study. Fracture healing is a very complex regenerative process that is affected by many factors, such as the patient's age, mechanical environment, and endocrine and local blood supply. Stress is a main factor, and some studies have shown that the strain of the PEEK external fixator under the same load is greater than that of the ordinary external fixator^31^. This is consistent with the conclusion that the curative effect and fracture healing time in the PEEK external fixator group in this study were superior to those in the common external fixator group. However, there are also studies showing that the application of PEEK materials for fracture fixation is relatively low, and the movement at the bone implant interface is increased, which is not conducive to the healing of fractures. Therefore, the effectiveness of PEEK materials needs to be studied further^2^.

There are reports on the application of PEEK materials in internal fixation^32^. However, there are few studies assessing the application of different external fixators for DRFs. The results of our research can provide a basis for further study. However, due to the small sample size, further research is needed to confirm the reliability of the data.

### *Conclusion*

There were no significant differences in the clinical and radiological results between the PEEK and titanium materials in the treatment of DRFs. However, the PEEK group was significantly better than the titanium material group in terms of the operation time and the frequency of fluoroscopy procedures, and this difference was statistically significant.

## Author Contributions

Mao Xie and Yinghao Cao contributed equally to this work and they are considered co‐first authors. Mao Xie, Zengwu Shao and Liming Xiong contributed to the study design; Yinghao Cao and Xianyi Cai performed the data collection and drafted the manuscript; Mao Xie and Yinghao Cao contributed to the statistical data analyses. Ke Nie and Liming Xiong are the correspondent authors. Mao Xie, Yinghao Cao, Ke Nie and Liming Xiong revised the manuscript. All authors contributed to interpretation of results and critical revisions of the manuscript; and they all agreed on its final appearance.
